# Cocaine in Teeth Extraction

**Published:** 1889-07

**Authors:** 


### Cocaine in Teeth-Extraction.

In the St. Petersburg weekly *Russkaia Meditzina*, No. 39, 1888, p. 623, Dr. I. S. Kolbasenko, of Kopal, writes that he resorted to Dr. G. Vian's method of local anæsthesia (*Therapeut. Monatschr.*, 1887, p. 502) in nine cases of teeth extraction, once in his own case. The method consists in injecting into the gum, on its buccal and lingual surfaces a solution of 4–5 of a grain of cocaine in ten drops of a 2 per cent, aqueous solution of carbolic acid. The following noteworthy conclusions have been arrived at by the author: 1. In the presence of an inflammatory swelling of the gum, cocaine does not develope any pain-killing effects whatever, which may be explained by the well-known fact that infiltrated tissues lose their absorptive power more or less completely. 2. But when the gum is normal, *i. e.*, not inflamed, the injection is invariably followed in a few seconds by a local numbness, and in two or three seconds, never later than in four minutes, by a complete anæsthesia. The extraction performed at this stage remains always quite painless from the beginning to the end. 3. The injection, however, is constantly accompanied by a train of more or less pronounced general phenomena, such as giddiness, clouding of sight, acceleration of the pulse, and, later on, by a kind of drunkenness with talkativeness, exhilaration, etc., the symptoms being especially marked in anæmic and nervous persons. In the latter a genuine hysterical fit, with clouded consciousness, tears, etc., may supervene. 4. Hence in those susceptible subjects a due caution is necessary. 5. The best means for preventing and weakening the general symptoms are these: (1) the injection should be preceded by the internal administration of a wine-glassful of rum or strong wine. (2) After the extraction the patient should be placed in a horizontal posture with his head hanging down. (3) Inhalation of two or three drops of amyl nitrite should be resorted to.

